# N_2_O Decomposition over Fe-ZSM-5: A Systematic Study in the Generation of Active Sites

**DOI:** 10.3390/molecules25173867

**Published:** 2020-08-25

**Authors:** Bryan Bromley, Chiara Pischetola, Linda Nikoshvili, Fernando Cárdenas-Lizana, Lioubov Kiwi-Minsker

**Affiliations:** 1 Department of Basic Sciences, Ecole Polytechnique Fédérale de Lausanne (GGRC-ISIC-EPFL), CH-1015 Lausanne, Switzerland; bromley.bryan@gmail.com; 2 Chemical Engineering, School of Engineering and Physical Sciences, Heriot Watt University, Edinburgh EH14 4AS, Scotland, UK; cp44@hw.ac.uk (C.P.); F.CardenasLizana@hw.ac.uk (F.C.-L.); 3 Regional Technological Centre, Tver State University, Zhelyabova Street, 33, 170100 Tver, Russia; nlinda@science.tver.ru

**Keywords:** FeZSM-5 activation, N_2_O decomposition, *α*-sites formation, transient response, NO and O_2_ temperature programmed desorption

## Abstract

We have carried out a systematic investigation of the critical activation parameters (i.e., final temperature (673–1273 K), atmosphere (He vs. O_2_/He), and final isothermal hold (1 min–15 h) on the generation of “*α*-sites”, responsible for the direct N_2_O decomposition over Fe-ZSM-5 (Fe content = 1200–2300 ppm). The concentration of *α*-sites was determined by (ia) transient response of N_2_O and (ib) CO at 523 K, and (ii) temperature programmed desorption (TPD) following nitrous oxide decomposition. Transient response analysis was consistent with decomposition of N_2_O to generate (i) “active” *α*-oxygen that participates in the low-temperature CO→CO_2_ oxidation and (ii) “non-active” oxygen strongly adsorbed that is not released during TPD. For the first time, we were able to quantify the formation of *α*-sites, which requires a high temperature (>973) treatment of Fe-ZSM-5 in He over a short period of time (<1 h). In contrast, prolonged high temperature treatment (1273 K) and the presence of O_2_ in the feed irreversibly reduced the amount of active sites.

## 1. Introduction

The release of nitrous oxide (N_2_O) into the environment was associated with stratospheric ozone depletion and global warming (global warming potential (GWP) 310 times greater relative to CO_2_) [1,2]. Although such releases have been reduced via environmental regulations, N_2_O is still generated through natural (e.g., nitrification/denitrification during microbial decomposition) and anthropogenic processes (e.g., manufacture of adipic and nitric acid, combustion of fossil fuels) [2,3]. N_2_O abatement technologies based on thermal decomposition and selective/non-selective catalytic reduction suffer from the associated complexity and high-energy consumption. Direct catalytic decomposition has emerged as a promising alternative for nitrous oxide emission control due to its simplicity, low energy requirements, and high efficiency. Direct N_2_O decomposition has been reported over bare oxides, hexaaluminates, spinels, and perovskites [2]. We have demonstrated previously [4] the viability of Fe-ZSM-5 for this process, where the redox properties of the iron species allow the decomposition of N_2_O at temperature >523 K. The process takes place on active sites called “*α*-sites”. These “*α*-sites” are able to accommodate adsorbed atomic oxygen (typically called “*α*-oxygen”) [5,6,7,8,9], which, in a second step, can (i) recombine forming O_2_, or (ii) react with other compounds (e.g., CO [4], CH_4_ [10], benzene [5,11], propane [12]). The formation of this active oxygen can be controlled by iron species, which, in turn, can be modified by the pre-treatment conditions (e.g., activation temperature, final isothermal hold time, and/or gas composition) [13,14,15,16,17], although the effect of each parameter remains a matter of debate. Indeed, catalyst activation by thermal treatment at high temperature can involve (i) calcination [18,19,20], (ii) steaming [11,13,21,22,23,24], or (iii) heating in an inert [25]/H_2_ stream [26] or under vacuum conditions [20], where exposure to different atmospheres has been shown to impact on the activity of Fe-ZSM-5 [20], attributed to changes in the amount of extra-framework iron species [27]. The position of Fe within the zeolite structure can be strongly influenced by the zeolite pre-treatment [26,28,29,30,31], while dealumination in the vicinity of Fe-sites [13,32,33,34], dehydroxylation of iron [25], and formation of defects [33,35] can also occur, with a direct impact on catalytic activity. Taking into consideration the complexity of the changes that can occur during the activation step, it is not surprising that the impact of high temperature treatment on N_2_O decomposition still remains largely unresolved. The deactivation in the presence of H_2_O/O_2_ can be useful to understand the structure of the *α*-sites and maximize their concentration, although this topic has not been considered to any significant extent.

In this work, we have set out a methodology based on “selective site titration” using a transient response (N_2_O followed by CO at 523 K) combined with temperature programmed desorption (TPD) directed at a systematic characterization (type, oxidation activity, and surface concentration) of oxygen species generated via direct N_2_O decomposition on the surface of Fe-HZSM-5. Our ultimate goal is to identify the activation and deactivation of the catalyst with a view to maximize the density of *α*-sites.

## 2. Results and Discussion

### 2.1. Effect of Activation Conditions on Formation of Active Sites

The effect of activation variables on the formation of active sites for HZSM-5_2300Fe_ was examined systematically by changing (i) the final temperature: 1273 K (high temperature, HT) vs. 973 K (low temperature, LT), (ii) atmosphere: He vs. 2% *v/v* O_2_/He, and (iii) final isothermal hold: 1 min vs. 15 min. The concentration of active sites determined using the different methods is shown in Table 1 and Table 2.

The transient N_2_O decomposition (C_N2_) provided the highest values in every instance, regardless of the final temperature, atmosphere, or final isothermal hold. The results obtained for thermal treatments in inert atmosphere (He) demonstrated a greater density of active sites for higher Fe-loading (2300 vs. 1200 ppm) at increased temperatures (HT vs. LT) with a maximum concentration attained after activation at 1273 K for 15 min. A decrease in the final activation temperature (to 973 K in He) resulted in a markedly lower concentration of active sites with only a marginal increase (ca. 10%) obtained for a prolonged activation period (2 h). This result demonstrates that formation of active sites for N_2_O decomposition is a high-barrier process and has a high-energy requirement.

The greater site concentration determined via N_2_O decomposition (vs. transient CO oxidation and TPD, Table 2) suggests that not all nitrogen released during the transient response of nitrous oxide is equivalent to one atomic oxygen adsorbed (see Equation (6) in Materials and Methods) [4]. The enhanced number of active sites obtained can be tentatively linked to the formation of NO with N_2_ release according to:

(1) N2O→NOad+12N2

where the transient N_2_O decomposition overestimates the quantity of active sites due to surface NO generation. Nonetheless, the concentration of NO (C_NO_, Table 2) is ca. two orders of magnitude lower than the amount of N_2_ released (C_N2_). This suggests that the excess measured by transient N_2_O decomposition may correspond to a fraction of oxygen adsorbed on the different type of sites generated during the activation step. These sites are not able to “spontaneously” change their oxidation state after nitrous oxide decomposition and do not contribute to the oxidation activity at low temperature. The concentration of sites able to adsorb non-active oxygen (denoted C_[O]_) can be estimated by subtraction (Equation (2)) using the other two methods (CO oxidation and TPD) and the results obtained are presented in Table 3.

(2) $$C_{\left[O\right]}=C_{N_{2}}-C_{O,TPD}$$

The results obtained demonstrate that C_[O]_ is sensitive to activation conditions with a measurable decrease at increase activation temperatures (973 vs. 1273 K) and in the presence of O_2_. We then considered the impact of activation temperature over the 673–773 K range with the same (60 min) final isothermal hold; the results obtained are shown in Table 4. It is interesting to note that the sites without catalytic properties (i.e., C_[O]_) were readily generated at low temperatures. In contrast, the formation of α-sites necessitates high temperatures with the almost exclusive generation of inactive sites during activation at 673 K (i.e., C_CO2_ and C_O,TPD_ ≤ 0.1 × 10^18^ sites g_catalyst_^−1^). This can be tentatively attributed to the mobility of oxygen on the zeolite surface, which can desorb during activation at 673 K, being replaced by atomic oxygen generated during the N_2_O decomposition at 523 K.

At activation for extended final isothermal hold time (i.e., 60 vs. 15 min), we observed a measurable increase in the number of inactive sites with activation temperature (Table 4), possibly linked to a facilitated dehydroxylation of the zeolite surface. In addition, the low concentration of CO_2_ (transient CO oxidation at 523 K) for samples activated at T ≥ 673 K (i.e., C_CO2_ ≤ 0.4 × 10^18^ sites g_catalyst_^−1^) suggests that there is a minimum temperature required for the formation of α-sites, which also depends on the activation time. A similar temperature dependence has been deemed necessary for the dehydroxylation of Fe [36], and therefore, this result suggests that α-sites are related to the presence of Fe. We have also addressed the impact of small amounts (2%) of oxygen in the inert feed during the activation at high-temperature. Under the same activation conditions, the results presented in Table 2 demonstrate a lower number of α-sites in the presence of oxygen. This can be tentatively attributed to the “destruction” of the active sites and is consistent with the further decrease (±4%) at prolonged hold times at the final activation temperature. It is important to note that the loss of active sites was irreversible: it was not possible to reach the same concentration of α-sites after a new high temperature treatment in inert atmosphere.

The results in this section prove that formation of α-sites is sensitive to the presence of Fe in ZSM-5 and requires zeolite activation at high temperature in an inert atmosphere.

### 2.2. N_2_O Adsorption

**Figure 1:**
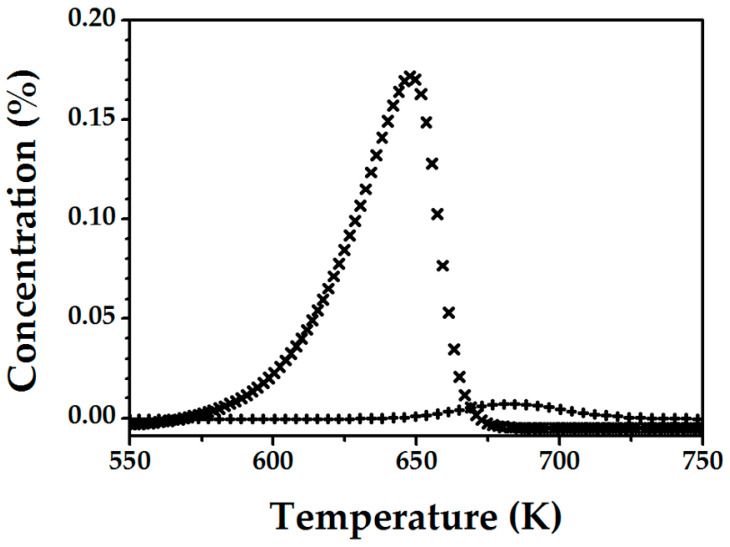
O_2_-(**✖**) and NO- (✚) TPD profiles for FeZSM-5_2300Fe_ activated in He at 1273 K for 30 min, after contact with N_2_O at 523 K for 5 min.

The FeZSM-5_2300Fe_ catalyst activated in He at 1273 K for 30 min was cooled to 523 K and saturated with atomic oxygen by N_2_O decomposition (at 523 K during 5 min). The accumulation of (O)_Fe,α_ and NO_ads_ was examined by TPD analysis and the results are presented in Figure 1.

It is known that NO exhibits a “co-catalytic effect” promoting the recombination of atomic oxygen [23,37,38]. The mechanism for N_2_O adsorption–decomposition has been reported previously [39], and can be schematically depicted as:

(3) $$N_{2}O_{gas}↔N_{2}O_{ad}\;(\mathrm{reversible}\;\mathrm{adsorption})\;\mathrm{instantaneous}$$

(4) $$N_{2}O_{ad}+{\left(\;\right)}_{Fe,\alpha }\to N_{2gas}+{\left(O\right)}_{Fe,\alpha }\;(\mathrm{deposition}\;\mathrm{of}\;\mathrm{oxygen})\;\mathrm{fast}$$

where (reversible) physical adsorption of N_2_O (Equation (3)) is followed by deposition of atomic oxygen on the catalyst surface and (molecular) nitrogen release (Equation (4)).

**Figure 2:**
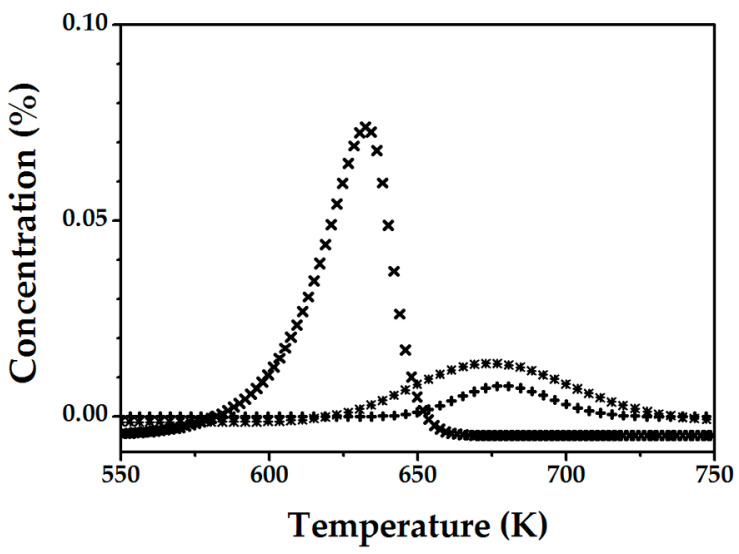
O_2_-(✖), NO-(✚) and N_2_O-TPD (✱) profiles for FeZSM-5_2300Fe_ activated in He at 973 K for 30 min, after contact with N_2_O at 523 K for 5 min.

A comparison of the TPD response for samples activated at 973 K (Figure 2) and 1273 K (Figure 1) revealed some differences. Indeed, for the sample activated at 973 K, a fraction of N_2_O adsorbed at 523 K and remained on the surface, being released at ca. 623–723 K during TPD (C_N2O, TPD_ = 0.61 × 10^18^ sites g_catalyst_^−1^, Table 5), a phenomenon not observed for the sample activated at high temperature (1273 K). This result suggests that generation of the (active) α-sites occurs in two steps, namely: (i) formation of the structure that allows chemisorption of N_2_O at 523 K and (ii) transformation of this structure into a site that allows a direct N_2_O decomposition with the generation of α-oxygen. This phenomenon can be related to the high coordinative unsaturation of Fe^2+^, a prerequisite for catalytic activity of iron, as often described in the literature [25,36]. The relationship between the activity of iron after high temperature activation and the presence of defects in the vicinity of iron has been demonstrated by infrared spectroscopy. The adsorption of NO after high temperature treatment has shown the presence of Fe(NO) with high frequency, indicative of the reduced electronic density of iron [33]. The results in Table 5 demonstrate the influence of the final isothermal hold time (for activation at 973 K) on the ability of the Fe-ZSME to adsorb N_2_O. The fresh (non-activated) sample exhibited no adsorption of N_2_O. After heating in He at 973 K, the sites for N_2_O adsorption were formed, where the concentration is sensitive to the final isothermal hold time, passing through the maximum at 15 min. After activation at 973 K for 2 h in He, the sample does not exhibit any capacity to adsorb N_2_O. This result suggests that the 2-step of active α-sites formation begins around this temperature. The adsorption of N_2_O without decomposition indicates an intermediate state between the formation of active α-sites and conversion of the initial inactive sites.

The positive effect of catalyst pre-treatment at high temperature has been often ascribed to the extraction of Fe from inactive to active extra-framework positions [30] as (–Fe(II)–O)_n_–Al species [40]. We envision a mechanism following two steps: (i) migration of iron from an inactive, ( )_Fe,inact_, to an active position (( )_Fe,N_2_O_) with subsequent (ii) formation of α-sites (probably due to loss of Fe coordination via dehydroxylation), which makes it able to stabilize oxygen and break the N_2_O molecule according to:

(5) $${\left(\;\right)}_{Fe,inact}\to {\left(\;\right)}_{Fe,N_{2}O}\to {\left(\;\right)}_{Fe,\alpha }$$

It is important to note that the transient response of N_2_O decomposition for a sample with a high concentration of sites able to adsorb N_2_O (sample activated at 973 K for 15 min) also exhibited a N_2_ signal before N_2_O with a shape similar to that of a sample containing only α-sites (sample activated at 1273 K for 60 min). This is indicative of a parallel formation at 973 K of the sites for N_2_O physical adsorption and for its decomposition, where once the active α-site has been created (like in the sample activated at 1273 K for 60 min), the N_2_O physical adsorption is no longer possible.

### 2.3. Degradation of Active Sites during High Temperature Treatment

**Figure 3:**
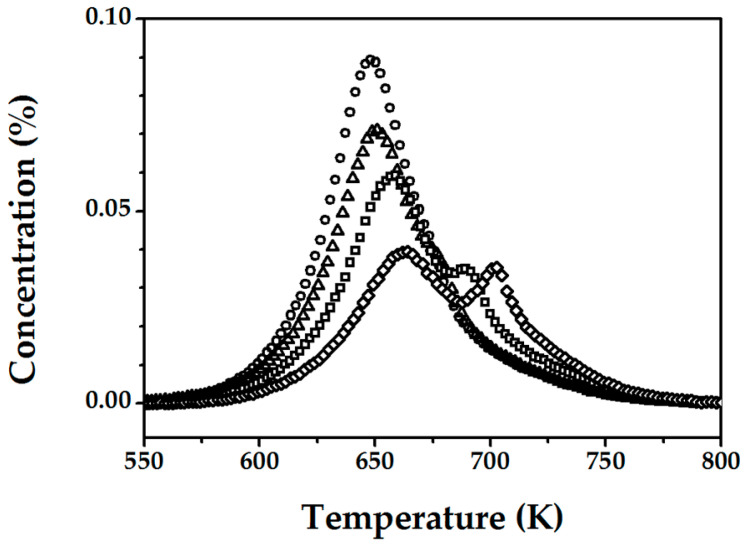
O_2_-TPD profiles for FeZSM-5_1200Fe_ activated in He at 1273 K for 1 (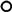), 3 (**△**), 5 (**☐**) and 15 h (**◇**), after contact with N_2_O at 523 K for 20 min.

**Figure 4:**
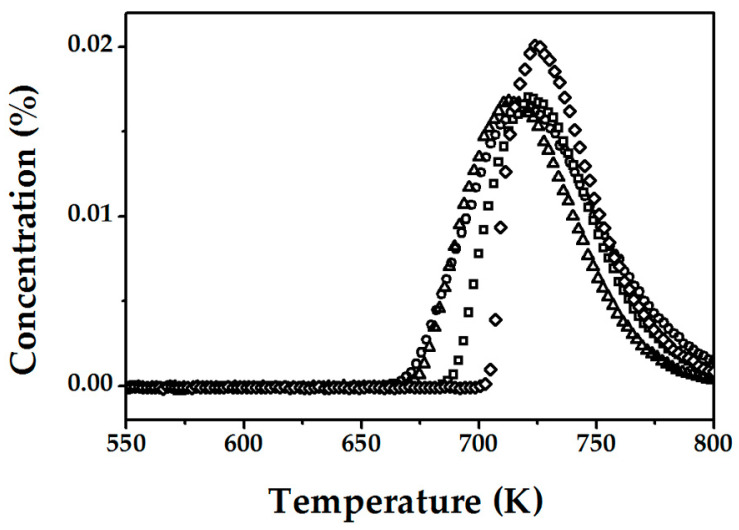
NO-TPD profiles for FeZSM-5_1200Fe_ activated in He at 1273 K for 1 (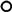), 3 (**△**), 5 (**☐**), and 15 h (**◇**) after contact with N_2_O at 523 K for 20 min.

The change in the number of active sites was measured at various times at the final isothermal hold during the activation of FeZSM-5_1200Fe_. The results from the three methods (transient N_2_O decomposition at 523 K, CO oxidation by the surface oxygen, and TPD) demonstrated a decrease in the total number of active sites during high temperature treatment (1273 K) with extended duration (1 h vs. 15 h). However, only TPD analysis has shown critical differences due to changes in the final isothermal hold time. TPD was carried out after the deposition of atomic oxygen from N_2_O (2% *v*/*v* N_2_O/He at 523 K for 20 min) for samples heated 1, 3, 5, and 15 h at 1273 K and the results in terms of oxygen and nitric oxide release are presented in Figure 3 and Figure 4, respectively.

We can see that treatment at high temperature and extended times appears to destroy active sites, where the amount of (O)_Fe,*α*_ obtained by TPD decreases (from C_O,TPD_ = 3.6 × 10^18^→2.3 × 10^18^ sites g_catalyst_^−1^) at increasing final isothermal hold times (1→15 h). Moreover, such a step is irreversible since it was not possible to attain the same concentration of active α-sites. The second observation is that the T_max_ for oxygen and NO desorption was shifted to higher values (for O_2_ from ca. 650 K for 1 h→ 675 K for 15 h and for NO from ca. 700 K to 720 K), a response consistent with a more energy demanding formation/desorption. This is in line with the work of Pirngruber et al. [41], who suggested that deposited oxygen is highly mobile and can migrate on the catalyst surface before recombination and release as O_2_. This migration is possible via peroxide bridges between two metals where the meeting of two bridges results in oxygen desorption. Several studies have demonstrated additional effects of treatment at high temperature including dealumination [13,33,42] and dehydroxylation of the zeolite lattice [18,35,43], where the latter is associated with the formation of oxygen vacancies that inhibits the generation of peroxide bridges, hampering the 2O→O_2_ step. In addition, a second peak for oxygen desorption (Figure 3) was observed at higher temperature for the samples treated for 5–15 h, indicative of a change in the mechanism of α-oxygen recombination/release. The simultaneous appearance of this high temperature (second) peak and decrease in the intensity of the low temperature (first) peak is clearly linked to the increase in final isothermal hold time and can be tentatively attributed to a modification of some of the active α-sites ( )_Fe,α_, resulting in a strong stabilization of the O that inhibits recombination.

## 3. Materials and Methods

### 3.1. Catalyst Preparation and Activation

Two HZSM-5_1200Fe_ (Si/Al = 120; Fe content = 1200 ppm) and HZSM-5_2300Fe_ (Si/Al = 42; Fe content = 2300 ppm) zeolites were prepared by hydrothermal synthesis according to a previously published procedure [40]. Briefly, tertraethyl orthosilicate (TEOS; Fluka, 98%) was added to an aqueous solution of tetrapropyl-ammonium hydroxide (TPAOH; Fluka, 20% in water) used as a template, sodium aluminate (NaAlO_2_; Riedel-de Haën, Na_2_O 40–45%, Al_2_O_3_ 50–56%), and Fe(NO_3_)_3_·9H_2_O (Fluka, 98%) under vigorous stirring; 0.8:0.1:0.016–0.032:33 mol ratio TEOS:TPAOH:NaAlO_2_:H_2_O and 160:3200 Si:Fe. The mixture was stirred (at room temperature) for 3 h and the gel obtained transferred to a stainless-steel autoclave lined with politetrafluoroetilene (PTFE) and kept at 453 K for two days. The product obtained was filtered, washed with deionized water and calcined (in air) at 823 K for 12 h. The solid was then converted into the H-form zeolite by exchange using an aqueous NH_4_NO_3_ solution (0.5 M). Catalyst activation was carried out to generate the *α*-sites (denoted ( )_Fe,α_ in this work) required for N_2_O decomposition (i.e., iron containing sites able to accommodate oxygen from N_2_O at 523 K to form an *α*-oxygen: (O)_Fe,α_). The activation step involved heating at 673–1273 K in 50 cm^3^ min^−1^ He and 2% *v/v* O_2_/He for 1–900 min. After activation, the samples were cooled down in He to 523 K.

### 3.2. Catalyst Characterization

Fe and Al content was measured by atomic absorption spectroscopy (AAS) using a Shimadzu AA-6650 spectrophotometer (Shimadzu Europe, Duisburg, Germany) with an air-acetylene flame from the diluted extract in 25% *v/v* HNO_3_/HCl. Transient response method and temperature programmed desorption (TPD) were employed to characterize the *α*-sites using the commercial Micromeritics AutoChem 2910 (Micromeritics Instrument Corporation, Gwinnett County, GA, USA) and Micromeritics AutoChem II units, equipped with a quartz plug-flow reactor. Product composition (i.e., 4 (He), 18 (H_2_O), 28 (N_2_, CO), 30 (NO), 32 (O_2_), 40 (Ar), 44 (N_2_O, CO_2_), and 46 (NO_2_)) was examined by mass spectrometric (MS) data analysis using a ThermoStar 200 (Pfeiffer Vacuum, Wetzlar, Germany) quadrupole mass spectrometer. Each experiment was repeated three times and the values quoted in this paper are the mean. Three methodologies that provide complementary information were employed to estimate the surface concentration of ( )_Fe,α_ and (O)_Fe,α_:

Transient N_2_O decomposition (at 523 K):

(6) $$N_{2}O+{\left(\;\right)}_{Fe,\alpha }\to N_{2}+{\left(O\right)}_{Fe,\alpha }$$

The absence of molecular oxygen in the gas phase suggests that each molecule of gaseous N_2_ is responsible for the adsorption of one oxygen atom on the catalyst surface. The concentration of active sites (i.e., amount of atomic oxygen deposited on the surface of the catalyst) can be obtained by N_2_ peak integration and is denoted “C_N2_”.

Transient CO oxidation (at 523 K):

(7) $$CO+{\left(O\right)}_{Fe,\alpha }\to CO_{2}+{\left(\;\right)}_{Fe,\alpha }$$

The transient N_2_O decomposition (Equation (7)) to saturate the catalyst surface with *α*-oxygen (i.e., (O)_Fe,α_) was followed by introduction of CO, which results in CO_2_ generation following Equation (7). The amount of CO_2_ formed (C_CO_2__) provides the number of *α*-sites containing atomic oxygen, active in low-temperature CO oxidation.

*TPD* (550–800 K):

(8) (O)Fe,α→ΔT()Fe,α+12O2

After saturation of the catalyst surface with *α*-oxygen through transient N_2_O decomposition (Equation (6)), molecular oxygen is desorbed via recombination during controlled thermal treatment (Equation (8)). The concentration of active sites responsible for O_2_ generation is denoted “C_O,TPD_” while “C_NO,TPD_” represents the NO formed according to Equation (1) and released during TPD.

In a typical experiment, the transient N_2_O decomposition was first carried out by flowing 20 cm^3^ min^−1^ of a 2%/2%/96% *v*/*v* N_2_O/Ar/He (Ar used as inert trace) mixture at 523 K for 5 min. After the decomposition of nitrous oxide (Equation (6)), the reactor was purged with 20 cm^3^ min^−1^ He for 10 min, and subjected to CO oxidation at 523 K (20 cm^3^ min^−1^ 35% *v/v* CO/He for 6 min; Equation (7)) or TPD (heating up to 800 K in 20 cm^3^ min^−1^ He at 30 K min^−1^; Equations (1) and (8)).

## 4. Conclusions

Quantitative transient methods of the determination of active sites have been used to study the influence of activation temperature, atmosphere, and time on the formation of “*α*-sites” in Fe-ZSM-5 catalysts (Fe 1200–2300 ppm) active in direct N_2_O decomposition. The heating in He at 673 K for 1 h led to the generation of sites able to decompose N_2_O at 523 K with the release of molecular nitrogen leaving behind oxygen on the zeolite surface. This surface oxygen was not able to react with CO at 523 K or desorb (as O_2_) during TPD to 800 K. In contrast, activation in He at 1273 K, even for a short period of time (1 min), led to the rapid formation of active sites able to decompose N_2_O to generate active *α*-oxygen (able to oxidize CO and recombine/desorb as O_2_ during TPD with a T_max_ ~640 K). The highest concentration of *α*-sites (up to 38% of total iron) was attained for activation in He at 1273 K during ca. 15 min, although a decrease in the density of *α*-sites was recorded at extended final isothermal hold time. The presence of molecular oxygen in the stream (2% O_2_ in He) also irreversibly reduced the number of *α*-sites. TPD analysis for the samples treated in He at 1273 K over prolonged times (3→15 h) revealed a reduction in the amount of molecular oxygen released, a shift of the O_2_ peak maxima to higher temperatures (640→670 K) and the appearance of a second desorption peak at higher temperature (690–710 K). This can be attributed to a reconstruction of *α*-Fe-sites that results in a stronger adsorption of atomic oxygen, with a higher energy demand for recombination/desorption as O_2_. To sum up, for the first time, we were able to quantitatively monitor the formation of *α*-sites during the activation of Fe-ZSM-5 at different temperatures, time, and atmosphere and demonstrate the highest concentration can be attained by a short heating treatment in He at 1273 K.

## Figures and Tables

**Table 1:** Characteristics of HZSM-5 catalysts after thermal treatment in He at 1273 K for 1 h.

Catalyst	Fe (ppm)	Si/Al	Site Concentration (10^18^ Sites g_catalyst_^−1^)		
			C_N_2__	C_CO_2__	C_O,TPD_
HZSM-5_1200Fe_	1200	120	3.5	3.2	3.1
HZSM-5_2300Fe_	2300	42	10.2	8.8	8.4

**Table 2:** Activation conditions and concentration of active sites for FeZSM-5_2300Fe_.

Atmosphere	Activation Temperature (K)	Final Isothermal Hold Time (min)	Site Concentration (10^18^ Sites g_catalyst_^−1^)						
			C_N_2__	C_CO_2__	% ^a^	C_O, TPD_	% ^b^	C_NO, TPD_	% ^c^
100% *v/v* He	973	1	4.4	2.4	55	1.8	40	0.04	1
		15	5.0	3.1	61	2.6	51	0.17	3
100% *v/v* He	1273	1	10.3	8.5	83	8.9	87	0.29	3
		15	10.7	9.4	88	9.5	89	0.31	3
2% *v/v* O_2_/He	1273	1	8.2	7.2	88	7.1	87	0.20	2
		15	7.4	6.4	86	6.2	83	0.18	2

^a^ (C_CO_2__/C_N_2__) × 100; ^b^ (C_CO, TPD_/C_N_2__) × 100; ^c^ (C_NO, TPD_/C_N_2__) × 100.

**Table 3:** Activation conditions and concentration of non-active oxygen (C_[O]_) after N_2_O decomposition (at 523 K) over FeZSM-5_2300Fe_.

Atmosphere	Activation Temperature (K)/Final Isothermal Hold (min)	C_[O]_ (10^18^ Sites g_catalyst_^−1^)	
		C_N_2__-C_CO_2__	C_N_2__-C_O,TPD_
100% *v*/*v* He	973/15	1.9	2.5
100% *v*/*v* He	1273/15	1.3	1.3
2% *v*/*v* O_2_/He	1273/15	1.1	1.2

**Table 4:** Activation conditions and concentration of actives sites (C_N_2__, C_CO_2__ and C_O,TPD_) and non-active oxygen (C_[O]_) after N_2_O decomposition (at 523 K) over FeZSM-5_2300Fe_.

Activation Temperature (K) ^a^/Final Isothermal Hold Time (min)	Site Concentration (10^18^ Sites g_catalyst_^−1^)					C_[O]_ (10^18^ Sites g_catalyst_^−1^)	
	C_N_2__	C_CO_2__	% ^b^	C_O,TPD_	% ^c^	C_N_2__-C_CO_2__	C_N_2__-C_O,TPD_
673/60	1.3	0.1	8	0	0	1.2	1.3
773/60	1.9	0.4	21	0.2	11	1.5	1.7

^a^ Activation in He; ^b^ (C_CO_2__/C_N_2__) × 100; ^c^ (C_CO, TPD_/C_N_2__) × 100.

**Table 5:** N_2_O adsorbed after chemisorption at 523 K (for 5 min) on FeZSM-5_2300Fe_.

Final Isothermal Hold Time (min) ^a^	N_2_O Adsorbed (10^18^ Sites g_catalyst_^−1^)
120	0
30	0.61
15	1.47
1	0.47

^a^ Activation in He at 973 K.
